# First-Line Pyrotinib Combination Therapy for HER2-Mutated Advanced NSCLC: A Retrospective Cohort Analysis

**DOI:** 10.3390/curroncol32030148

**Published:** 2025-03-04

**Authors:** Yan Xiang, Meiling Zhang, Qian Wang, Jingwen Liu, Lulin Zeng, Ao Sun, Kaihua Lu

**Affiliations:** Department of Oncology, The First Affiliated Hospital of Nanjing Medical University, Nanjing 210029, China

**Keywords:** human epidermal growth factor receptor 2, non-small-cell lung cancer, pyrotinib, combination therapy, safety

## Abstract

**Background:** HER2 mutations are rare driver events in advanced NSCLC, with limited relief from current targeted therapies. This study aimed to characterize the molecular features of HER2-mutant NSCLC and to evaluate the clinical efficacy of pyrotinib-based combination therapy as a first-line treatment, providing evidence for optimizing treatment strategies. **Methods:** NSCLC patients diagnosed at Jiangsu Province People’s Hospital from 2016 to 2024 were enrolled. HER2-positive cases were screened by IHC/FISH and further profiled by NGS. Treatment response was assessed by RECIST 1.1, and survival analysis was performed using Kaplan–Meier and log-rank tests. **Results:** Among 144 HER2-mutant NSCLC cases confirmed by NGS, 10 insertion mutations, 26 missense mutations, and 2 fusion mutations were identified. The most common mutation was the exon 20 p.A775_G776insYVMA (47.9%), and TP53 was the most frequent co-mutation (10.4%). In terms of efficacy, the pyrotinib-based combination therapy demonstrated significant clinical benefit, with an ORR of 33.3%, DCR of 95.2%, median PFS (mPFS) of 11.3 months (95% CI: 10.27–12.26), and median OS (mOS) of 21.0 months (95% CI: 18.00–23.94). Subgroup analysis revealed no significant impact of mutation subtype or co-mutation status on the treatment efficacy, but patients with brain metastases had a significantly worse prognosis than those without metastasis (mPFS: 5.1 vs. 12.9 months, *p* < 0.01; mOS: 9.3 vs. 26.5 months, *p* < 0.01). All TRAEs were grade 1–3 (any grade: 90.5%; grade 3: 14.3%), with the most common TRAE being diarrhea (any grade: 85.7%; grade 3: 9.5%). **Conclusions:** Pyrotinib-based combination therapy is a feasible first-line treatment for HER2-mutant NSCLC, demonstrating significant survival benefits and manageable toxicity. However, brain metastasis patients require enhanced comprehensive management.

## 1. Introduction

Non-small-cell lung cancer (NSCLC) accounts for 80% to 85% of all lung cancer cases [1]. In 2022, lung cancer ranked first in both incidence and mortality among malignant tumors in China. For advanced-stage patients, molecular targeted therapy has significantly improved prognosis, and human epidermal growth factor receptor 2 (HER2), as an emerging therapeutic target, has attracted considerable attention [2,3]. HER2 gene mutations (2% to 4%), amplifications (2% to 5%, and the proportion can increase to 10% after epidermal growth factor receptor tyrosine kinase inhibitor (EGFR-TKI) resistance), and protein overexpression (2% to 30%) are the main activation modes [4,5]. The heterogeneity of these alterations leads to significant differences in clinical prognosis [6]. Notably, HER2 mutation patients have a high incidence of brain metastases, and traditional chemotherapy is limited in efficacy, especially with the A775_G776insYVMA insertion mutation (which accounts for 80% of the mutations in exon 20), which poses significant treatment challenges [7].

Currently, among HER2-targeted drugs, pan-HER2 TKIs (such as afatinib) have an objective response rate (ORR) of less than 20%, and the chemotherapy regimen based on trastuzumab does not show better efficacy compared to chemotherapy alone [median progression-free survival (mPFS): 6.1 months vs. 7 months] [8,9,10,11]. However, novel irreversible TKIs (such as pyrotinib) and antibody–drug conjugates (ADCs, such as trastuzumab–deruxtecan, DS8201) have demonstrated breakthrough efficacy [12,13,14]. Pyrotinib is an irreversible pan-HER inhibitor independently developed in China, which permanently binds to the ATP-binding sites of EGFR/HER1, HER2, and HER4 to block dimerization and downstream signaling, thereby inhibiting tumor growth. In phase II multi-center clinical trials, pyrotinib showed a higher objective response rate (ORR), disease control rate (DCR), and PFS in patients with HER2 insertion mutations, especially the YVMA subtype, and has been proven to be a promising HER2-TKI. In the 2021 Chinese Society of Clinical Oncology (CSCO) NSCLC treatment guidelines, pyrotinib was listed as the only TKI targeting HER2 mutations. The CTONG1702 study further confirmed that in rigorously selected first-line patients (*n* = 28), pyrotinib treatment significantly extended mPFS (7.3 vs. 3.0 months) and increased ORR by more than threefold (35.7% vs. 0%), highlighting its clinical advantage [15].

This study focused on 21 advanced-stage HER2-mutated NSCLC patients who received first-line treatment with a pyrotinib-based regimen from a cohort of 144 HER2-mutated NSCLC patients at Jiangsu Province People’s Hospital. We systematically analyzed its efficacy and safety and explored the correlation between different mutation subtypes and therapeutic responses, aiming to provide evidence-based insights for optimizing precision therapeutic strategies.

## 2. Materials and Methods

### 2.1. Patient Selection

This study was a single-center retrospective cohort study, which included NSCLC patients diagnosed by pathology at Jiangsu Province People’s Hospital from May 2016 to May 2024. HER2-positive cases (*n* = 144) were initially screened by IHC/FISH and further validated for HER2 mutations using next-generation sequencing (NGS). We systematically analyzed their clinicopathological features and molecular mutation profiles. The efficacy analysis focused on patients who met the following criteria (*n* = 21): ① age ≥18 years; ② AJCC 8th edition staging for stage IIIB-IV inoperable advanced/metastatic NSCLC; ③ diagnosed with HER2 driver mutations via NGS and had not received prior systemic treatment for metastatic disease (previous neoadjuvant/adjuvant chemotherapy or radiotherapy was allowed, but the interval between the last treatment and the diagnosis of metastasis was ≥6 months); ④ first-line treatment with a pyrotinib-based chemotherapy regimen; ⑤ ECOG performance status 0–2 and complete clinical data. This study was approved by the Ethics Committee of the First Affiliated Hospital of Nanjing Medical University.

### 2.2. Method

IHC: Staining was performed using a standardized IHC kit (Dako HER2 rabbit monoclonal antibody), and the interpretation standards were referenced from the “Breast Cancer HER2 Testing Guidelines (2019 Edition)” with extended application to NSCLC. HER2 protein overexpression was defined as IHC 3+ or IHC 2+ with positive FISH validation.

FISH: HER2 amplification was detected using the HER2/CEP17 dual-probe kit (Jiangsu Weizhen Biomedical Technology Co., Ltd., Suzhou, China). The interpretation strictly followed the 2018 ASCO/CAP guidelines. Positive results were defined by any of the following conditions: ① HER2/CEP17 ratio ≥ 2.0 and average HER2 copy number ≥ 4.0 signals/cell; ② HER2/CEP17 ratio < 2.0 but average HER2 copy number ≥ 6.0 signals/cell.

NGS: Targeted sequencing was performed using the Illumina HiSeq platform (Geneseeq, Nanjing, China), covering all exons of the HER2 gene and adjacent splice regions. Sequencing depth reached 1000× (tumor tissue), with a sensitivity threshold of 1% allele frequency. Variant identification was based on the GATK 4.1 standard workflow, and clinical significance was annotated using the COSMIC and ClinVar databases.

### 2.3. Response Assessment

Patients received first-line treatment with platinum-based chemotherapy combined with pyrotinib (400 mg/day orally, adjusted to 320 mg/day in case of dose intolerance, Jiangsu Hengrui Pharmaceuticals Co., Ltd., Lianyungang, China). Efficacy was assessed every 2 cycles (8 weeks) through CT/MRI imaging (independent radiological assessment, Response Evaluation Criteria in Solid Tumors 1.1 (RECIST 1.1) criteria) and laboratory indicators. The primary observation endpoints were PFS and OS, while secondary endpoints included ORR, DCR, and treatment-related adverse events (TRAEs, graded according to Common Terminology Criteria for Adverse Events Version 5.0 (CTCAE 5.0)). Follow-up was conducted until 31 August 2024, recording disease progression, death, or ≥grade 3 intolerable toxic events.

### 2.4. Statistical Analysis

Data analysis was performed using SPSS 26.0 and GraphPad Prism 8.0. The Kaplan–Meier method was used to assess survival, and the log-rank test was applied to analyze subgroup differences. *p* < 0.05 was considered statistically significant.

## 3. Results

### 3.1. Clinical Characteristics

A total of 144 HER2-mutant NSCLC patients were enrolled in this study, including 57 males (39.6%) and 87 females (60.4%), with an average age of 62.0 ± 10.0 years. The proportion of patients with a history of smoking was 26.4% (38/144). The predominant pathology was adenocarcinoma (140 cases, 97.2%), with only 4 cases of non-adenocarcinoma (3 squamous cell carcinoma and 1 adenosquamous carcinoma). Clinical staging showed that 76.4% (110/144) of patients were diagnosed with stage III–IV disease at the initial presentation. ECOG scores of 0–1 were observed in 81.9% (95/116) of patients, and 16.7% (24/144) had concomitant brain metastasis.

For the 21 patients with advanced adenocarcinoma (all stage IIIB-IV) who received pyrotinib combined with chemotherapy, the statistics showed that 71.4% (15/21) were male, with an average age of 66.3 ± 9.4 years, and 42.9% (9/21) had a smoking history. Among them, 81.0% (17/21) had an ECOG score of 0–1, and the brain metastasis rate at initial diagnosis was higher than the overall cohort (33.3% vs. 16.7%). Detailed clinical characteristics of all patients are shown in Table 1.

### 3.2. Molecular Characteristics

**Figure 1:**
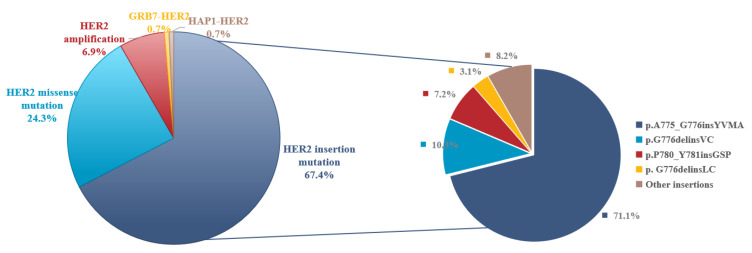
Molecular characteristic of HER2 mutations. (Percentages in the right part of Figure 1 total 99.9% due to rounding).

This study conducted a molecular characteristic analysis of 144 patients with HER2-activating mutations. The mutations were primarily located in the tyrosine kinase domain (TKD), followed by the extracellular domain (ECD) and the transmembrane domain (TMD). A total of 38 HER2 mutation subtypes were identified, including 10 insertion mutations (97 cases, 67.4%), 26 missense mutations (35 cases, 24.3%), 2 fusion mutations (2 cases, 1.4%), and 10 cases of primary HER2 amplification (6.9%) (Figure 1). Among these, exon 20 was the core hotspot for insertion mutations, with the following predominant mutations: A775_G776insYVMA (69 cases, 47.9%), G776delinsVC (10 cases, 6.9%), P780_Y781insGSP (7 cases, 4.9%), G776delinsLC (3 cases, 2.1%), and other types (8 cases, 5.6%) (Table 2). In the missense mutations, S310F (five cases, 3.5%), V659E (four cases, 2.8%), and L755 site mutations (L755S/L755A/L755P, two cases each) were notable. Additionally, 17 rare single-point mutations were observed (such as L785F, V777L, R1230Q, G776V, D1144H, S310Y, V842I, G727A, L755A, Y803N, R896G, Q24H, G776S, A678Q, R811L, I655V, and P1170A), along with 2 fusion mutations. Among them, HAP1-HER2 is reported for the first time, while GRB7-HER2 has been associated with poor prognosis in colorectal cancer.

Among the 31 patients with evaluable co-mutations, TP53 mutations were the most common (48.4%, 15/31), followed by 12.9% (4/31) of patients having HER2 double mutations (e.g., V777_G778insCG + G776R, A775_G776insYVMA + Q24H, etc.), and another 12.9% (4/31) with primary HER2 amplification. Other co-occurring abnormalities included KRAS mutations (9.7%, 3/31, including G12A/G12V/G15S subtypes) and 16.1% (5/31) with cell cycle regulation pathway gene alterations (such as MDM2, MDM4, MYC, and RB1). These results suggest that HER2-mutant tumors exhibit significant heterogeneity, and their genetic mutation profiles and co-mutation patterns may have a crucial impact on targeted therapy strategies.

### 3.3. Efficacy Analysis

**Figure 2:**
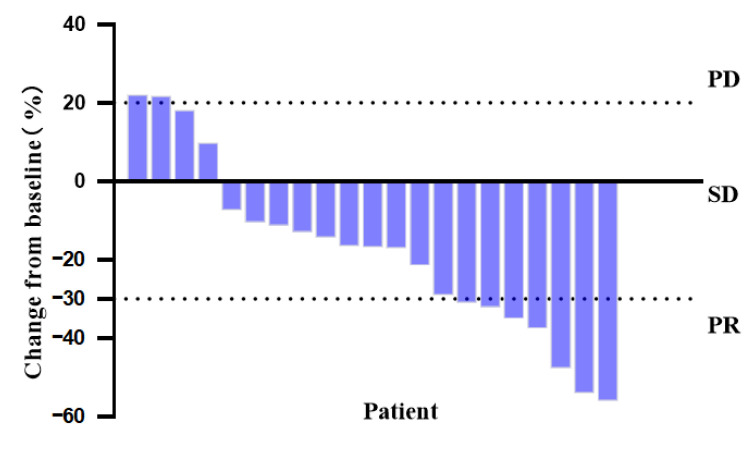
Treatment response and its proportions for 21 patients.

**Figure 3:**
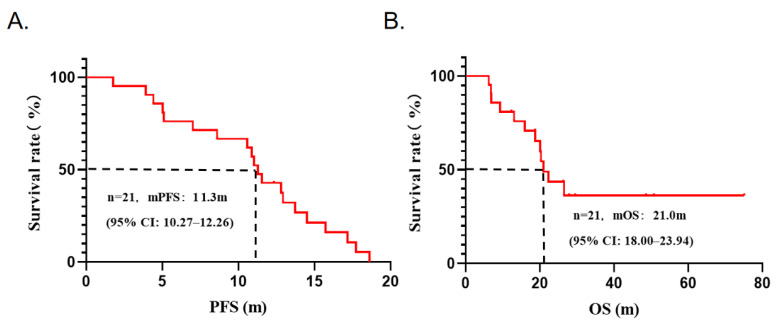
Survival curves of 21 patients treated with pyrotinib combined with chemotherapy. (**A**): PFS; (**B**): OS.

As of 31 August 2024, the median follow-up time was 29.5 months (95% CI: 25.4–33.7). Among the 21 patients with stage IIIB-IV NSCLC, 9 patients (42.9%) were still receiving treatment and 12 patients (57.1%) had died. Imaging assessments showed partial response (PR) in 7 patients (33.3%), stable disease (SD) in 13 patients (61.9%), and disease progression (PD) in 1 patient (4.8%) (Figure 2). The ORR was 33.3%, and the DCR was 95.2%. Survival analysis revealed an mPFS of 11.3 months (95% CI: 10.27–12.26) and an mOS of 21.0 months (95% CI: 18.00–23.94). The clinical information and survival curves of these patients were presented in Table 3 and Figure 3. These findings indicate that pyrotinib-based combination therapy is a promising first-line treatment option for HER2-mutant NSCLC, demonstrating significant survival benefits.

### 3.4. Subgroup Analysis

**Figure 4:**
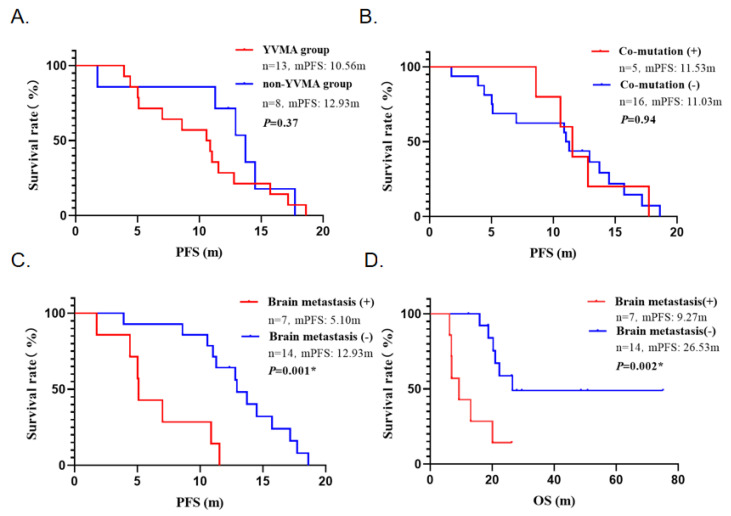
Kaplan–Meier survival curves showing PFS and OS in different groups. (**A**) Mutation subtypes and PFS: No significant difference between YVMA and non-YVMA groups (*p* = 0.37). (**B**) Co-mutation and PFS: No significant difference between positive and negative groups (*p* = 0.94). (**C**) Brain metastasis and PFS: Significantly lower in patients with brain metastasis (*p* = 0.001). (**D**) Brain metastasis and OS: Significantly lower in patients with brain metastasis (*p* = 0.002). (* indicates a statistically significant difference in *p*-value.)

Among the 21 patients receiving treatment, 14 patients carried the A775_G776insYVMA mutation (YVMA group), and the remaining 7 patients had other mutation types (non-YVMA group). Although the YVMA group showed a trend toward shorter PFS (mPFS: 10.57 months vs. 13.73 months), the log-rank test analysis indicated that the difference between the two groups was not statistically significant (*p* = 0.37). Further analysis of the impact of co-mutation status showed that patients with co-mutations (mPFS:11.53 months, 95% CI: 9.47–13.61) had comparable survival benefit to those with only HER2 mutations (mPFS:11.03 months, 95% CI: 10.25–11.82) (*p* = 0.94) (Figure 4A,B).

It is noteworthy that brain metastasis status significantly impacted clinical outcomes. Patients with brain metastasis showed poorer survival outcomes (mPFS: 5.10 months, 95% CI: 4.93–5.27; mOS:9.27 months, 95% CI: 3.28–15.26), significantly lower than those without brain metastasis (mPFS:12.93 months, 95% CI: 11.37–14.50; mOS: 26.53 months, 95% CI: NA-NA). Statistical analysis revealed significant differences between the two groups (*p* < 0.01) (Figure 4C,D).

### 3.5. Safety

In this study, among the 21 patients with HER2-mutant advanced NSCLC, the incidence of a TRAE of any grade was 90.5% (19/21), with predominantly grade 1–2 mild reactions. No grade 4 or higher severe TRAEs were observed. The most common adverse events were diarrhea (85.7%, 18/21), followed by bone marrow suppression (52.4%, 11/21), nausea (47.6%, 10/21), fatigue (42.9%, 9/21), and elevated transaminases (28.6%, 6/21). Grade 3 TRAEs occurred in only three cases (14.3%), including two cases of diarrhea (9.5%) and one case of bone marrow suppression (4.8%) (Table 4). Two patients required a pyrotinib dose reduction to 320 mg due to intolerable grade 3 diarrhea. Notably, none of the patients discontinued treatment due to severe adverse events or treatment-related death, indicating that the pyrotinib combination regimen has a controllable safety profile in this patient population.

## 4. Discussion

Previous studies have shown that HER2 mutations exhibit significant heterogeneity, with most being single mutations [6]. In this retrospective study, a total of 38 different HER2 mutation subtypes were identified, including 10 insertion mutations (97 cases, 67.4%), 26 missense mutations (35 cases, 24.3%), and 2 fusion mutations (2 cases, 1.4%). Among them, HER2 insertion mutations almost exclusively occurred in exon 20, which is consistent with the proportion reported in other studies [16]. Additionally, we identified other less common HER2 mutation subtypes, such as missense mutations in exons 1, 8, 17, 18, and 27. Furthermore, NGS detected a GRB7-HER2 fusion in the tissue sample of a 74-year-old patient with advanced adenosquamous lung cancer. There have been case reports suggesting that this fusion is associated with poor prognosis in colorectal cancer patients, with rapid disease progression [17]. However, no related drug reports have been found for lung cancer. In this study, the patient initially received platinum-based chemotherapy, but multiple metastatic lesions progressed after 5 months. The patient refused further chemotherapy and started second-line oral pyrotinib (400 mg daily). After 4 months, the disease progressed, and the patient switched to third-line immunotherapy. On 10 February 2023, the patient passed away due to severe pneumonia, with an OS of 15 months.

In the context of emerging anti-HER2 therapies for NSCLC, our findings are consistent with recent advancements while highlighting distinct advantages. In the CTONG1702 study [15], the ORR for first-line pyrotinib monotherapy was 35.7%, with an mPFS of 7.3 months and an mOS of 14.3 months. In contrast, our study showed that the mPFS for the combination of pyrotinib and chemotherapy (11.3 months) was significantly superior to that of monotherapy, suggesting that combination therapy may improve efficacy. The phase II DESTINY-Lung01 trial [18] demonstrated that T-DXd achieved an mPFS of 8.2 months and an mOS of 17.8 months in HER2-mutant NSCLC patients as a second-line and later treatment. However, in our study, the pyrotinib–chemotherapy combination as first-line treatment achieved superior outcomes (mPFS of 11.3 months; mOS of 21.0 months). This difference may reflect variations in treatment stages and patient population characteristics, although our retrospective design warrants cautious interpretation. Furthermore, compared to T-DXd, the pyrotinib-based regimen appears to have advantages in terms of safety and tolerability, particularly with a lower incidence of grade ≥ 3 interstitial lung disease (ILD) (0% in our cohort vs. 6.6% with T-DXd) [18].

It is noteworthy that HER2-TKIs show varying efficacy profiles. For example, the efficacy of pan-HER2 TKIs such as Afatinib is not significant, with an ORR ranging from 0% to 20%, and the mPFS being from 2.8 to 3.7 months [10,11]. In the ZENITH20-2 cohort, the mPFS for poziotinib was reported to be only 5.5 months [14]. Beamion LUNG-1 is an ongoing, first-in-human, open-label study evaluating the safety and efficacy of zongertinib (a selective covalent HER2 TKI) [19] in HER2-mutant NSCLC patients (Stage IB). According to the latest results from ESMO 2024 [20], zongertinib showed an ORR of 44%, a DCR of 93%, and a 6-month PFS rate of 69%. Its efficacy outperforms traditional non-selective TKIs (such as afatinib), likely due to zongertinib’s higher selectivity and potent inhibition of HER2 exon 20 insertion mutations. Although our pyrotinib combined with chemotherapy regimen showed an ORR of 33.3%, its overall efficacy still holds certain competitive advantages, particularly in terms of DCR (95.2%) and mPFS (11.3 months). The combination treatment regimen may overcome the resistance issues associated with single-agent targeted therapy through synergistic effects, thereby providing sustained disease control. Although these cross-trial comparisons are influenced by heterogeneity in study design, our data suggest that pyrotinib combined with chemotherapy could be a clinically meaningful option for treating HER2-mutant NSCLC. Future randomized trials are needed for prospective validation to confirm the optimal first-line treatment for advanced HER2-mutant NSCLC patients.

Existing studies have shown that advanced NSCLC with the HER2 exon 20 YVMA insertion mutation has a poorer prognosis compared to other HER2 mutation types and exhibits limited efficacy to both chemotherapy and targeted therapies. Yang S and colleagues [21] found that, among 82 patients receiving first-line chemotherapy, the YVMA insertion mutation was associated with a higher incidence of brain metastasis and poorer chemotherapy outcomes compared to non-YVMA mutations (mPFS: 5.23 months vs. 7.73 months; ORR: 30.9% vs. 51.9%). In the study by Fang W and colleagues [22], patients with the A775_G776insYVMA mutation showed worse efficacy to targeted therapy, with an ORR of 0%, a DCR of 35.7%, and a PFS of only 1.2 months. In contrast, patients with G778_P780dup or G776delinsVC mutations had an ORR of 40%, a DCR of 100%, and a PFS of 7.6 months. These results suggest that different HER2 mutation types may have a significant impact on treatment outcomes. However, in this study, we explored the pyrotinib combined with chemotherapy treatment regimen, which showed significant efficacy in both YVMA mutation and non-YVMA mutation patients. The log-rank test revealed that the PFS for the YVMA group and non-YVMA group were 10.56 months and 12.93 months, respectively. Although no statistical difference was observed (*p* = 0.50), the combined regimen demonstrated better overall efficacy than traditional treatments. Notably, under the pyrotinib combined chemotherapy treatment model, HER2 mutation subtypes (including the YVMA insertion mutation) and co-mutation status did not significantly affect treatment outcomes, suggesting that this regimen may have broader applicability. Given the limited sample size, future studies with larger sample sizes are needed to further validate this conclusion.

Several limitations of this study should be acknowledged. First, as a single-center retrospective study, in addition to the risk of selection bias, it is important to note the absence of a pyrotinib monotherapy group, which prevents the quantification of chemotherapy’s contribution to the combination regimen, thus limiting the accurate evaluation of pyrotinib’s independent efficacy. Second, the current study design cannot distinguish whether the advantage of the combination regimen comes from synergistic effects or merely from dose addition. Future studies should consider conducting a three-arm randomized controlled trial (pyrotinib monotherapy vs. chemotherapy monotherapy vs. combination therapy) and include pharmacokinetic assessments to clarify the interaction mechanisms. Third, compared to T-DXd, although the combination regimen in this study showed superior PFS, the ORR was relatively low (33.3% vs. 55%), suggesting that the optimization of the dosing regimen or the exploration of new combination strategies may be needed. Finally, due to the relatively small sample size, the specific role of radiotherapy in the pyrotinib treatment process was not further explored. Based on the NCCN guidelines recommending local interventions for oligometastatic lesions (Level 1 evidence), future studies could further explore the synergistic effects of pyrotinib combined with radiotherapy.

## 5. Conclusions

In conclusion, pyrotinib-based combination therapy may become a promising option for the first-line treatment of HER2-mutant NSCLC. Its significantly prolonged PFS and outstanding DCR have preliminarily confirmed its clinical value, and its overall safety profile is manageable. It is particularly important to note that the treatment response in patients with central nervous system metastasis still requires optimization through multidisciplinary intervention strategies. Future phase III trials are needed to validate its survival benefits and explore combination therapies with ADCs, radiotherapy, or precision strategies targeting specific mutation subtypes.

## Figures and Tables

**Table 1:** Clinical characteristics of all patients and patients included in the treatment.

Characteristics	All Patients	Pyrotinib Plus Chemotherapy
**Total**	144	21 (43.8%)
**Gender**		
Female	87 (60.4%)	15 (71.4%)
Male	57 (39.6%)	6 (28.6%)
**Age**	62.0 ± 10.0	66.3 ± 9.4
**Smoking history**		
No	106 (73.6%)	12 (57.1%)
Yes	38 (26.4%)	9 (42.9%)
**Physiology**		
Adenocarcinoma	140 (97.2%)	21 (100.0%)
Squamous cell carcinoma	3 (2.1%)	0 (0.0%)
Adenosquamous carcinoma	1 (0.7%)	0 (0.0%)
**TNM Stage**		
I–II	34 (23.6%)	0 (0.0%)
III–IV	110 (76.4%)	21 (100.0%)
**ECOG score**		
0–1	95 (81.9%)	17 (81.0%)
≥2	21 (18.1%)	4 (19.0%)
**Brain metastasis**		
No	120 (83.3%)	14 (66.7%)
Yes	24 (16.7%)	7 (33.3%)

**Table 2:** HER2 exon20 insertion subtypes and proportion.

HER2 exon20 Insertion Subtypes	Number	Proportion
p.A775_G776insYVMA	69	47.90%
p. G776delinsVC	10	6.90%
p.P780_Y781insGSP	7	4.90%
p. G776delinsLC	3	2.10%
p. G776delinsVV	2	1.40%
p.G778_S779insCPG	2	1.40%
p.G776_V777delinsCVC	1	0.70%
p.G776_V777delinsAVCG	1	0.70%
p.V777_G778insCG	1	0.70%
p.M774delinsVWL	1	0.70%
**Total**	97	67.4%

**Table 3:** Clinical information of 21 HER2 mutant advanced NSCLC patients.

Case	Gender/ Age	Brain Metastases	HER2 Mutation Subtype	Co-Current Genes	First-Line Treatment	Efficacy	PFS/Months	OS/Months
1	M/76	Yes	Exon20 p.A775_G776insYVMA	/	Pyrotinib/Pemetrexed/Carboplatin	SD	5.03	6.83
2	M/82	No	Exon8 p.S310F	/	Pyrotinib/Pemetrexed/Carboplatin	PR	14.50	50.73+
3	M/74	No	Exon20 p.A775_G776insYVMA	/	Pyrotinib/Pemetrexed/Carboplatin	SD	17.17	27.87+
4	F/83	No	Exon20 p.A775_G776insYVMA	/	Pyrotinib/Pemetrexed/Carboplatin	SD	3.90	15.93
5	F/73	No	Exon20 p.A775_G776insYVMA	TP53	Pyrotinib/Pemetrexed/Carboplatin	SD	10.57	20.30
6	F/65	Yes	Exon20 p.A775_G776insYVMA	/	Pyrotinib/Nab-paclitaxel/Carboplatin	SD	10.87	26.27+
7	F/67	Yes	Exon20 p.A775_G776insYVMA	/	Pyrotinib/Pemetrexed/Carboplatin	PR	5.10	6.23
8	M/56	No	Exon 20 p.S783A	/	Pyrotinib/Pemetrexed/Carboplatin	SD	13.73	18.77
9	M/60	Yes	Exon20 p.A775_G776insYVMA	/	Pyrotinib/Pemetrexed/Carboplatin	PR	7.00	13.03
10	F/67	No	Exon20 p.G776delinsVC	/	Pyrotinib/Pemetrexed/Carboplatin	SD	11.27	26.53
11	F/67	No	Exon20 p.A775_G776insYVMA	TP53	Pyrotinib/Pemetrexed/Carboplatin	SD	12.80	75.10+
12	F/67	No	Exon20 p.A775_G776insYVMA	TP53	Pyrotinib/Pemetrexed/Carboplatin	SD	8.60	22.30
13	F/47	No	Exon20 p.A775_G776insYVMA	/	Pyrotinib/Pemetrexed/Carboplatin/Bevacizumab	SD	18.60	18.67+
14	F/64	No	Exon20 p.G778_S779insCPG	MDM2	Pyrotinib/Pemetrexed/Carboplatin/Bevacizumab	PR	17.73	48.60+
15	F/63	Yes	Exon20 p.A775_G776insYVMA	TP53	Pyrotinib/Pemetrexed/Carboplatin/Bevacizumab	PR	11.53	20.13
16	F/59	Yes	Exon19 p.L755S	/	Pyrotinib/Pemetrexed/Carboplatin/Bevacizumab	PD	1.77	9.27
17	F/58	No	Exon20 p.A775_G776insYVMA	/	Pyrotinib/Paclitaxel liposome/ Carboplatin/Bevacizumab	SD	11.03	20.97
18	F/50	No	Exon19 p.L755A	/	Pyrotinib/Pemetrexed/Carboplatin/Bevacizumab	PR	12.33+	12.33+
19	F/76	Yes	Exon20 p.A775_G776insYVMA	/	Pyrotinib/Pemetrexed/Carboplatin/Bevacizumab	SD	4.40	6.93
20	F/71	No	Exon17 p.I655V	/	Pyrotinib/Pemetrexed/Carboplatin/Bevacizumab	SD	12.93	29.53+
21	M/67	No	Exon20 p.A775_G776insYVMA	/	Pyrotinib/Pemetrexed/Carboplatin	PR	15.73	26.27+

**Table 4:** Treatment-related adverse events in 21 patients.

Adverse Events	Any Grade	Grade 1–2 (%)	Grade 3 (%)
Any events	19 (90.5%)		
Diarrhea	18 (85.7%)	16 (76.2%)	2 (9.5%)
Myelosuppression	11 (52.4%)	10 (47.6%)	1 (4.8%)
Nausea	10 (47.6%)	10 (47.6%)	0 (0.0%)
Fatigue	9 (42.9%)	9 (42.9%)	0 (0.0%)
Decreased appetite	7 (33.3%)	7 (33.3%)	0 (0.0%)
Elevated transaminase	6 (28.6%)	6 (28.6%)	0 (0.0%)
Electrolyte imbalance	3 (14.3%)	3 (14.3%)	0 (0.0%)
Hand-foot syndrome	2 (9.5%)	2 (9.5%)	0 (0.0%)

## Data Availability

The data presented in this study are openly available in FigShare at [doi: 10.6084/m9.figshare.28190504], reference number [28190504]. We declare that all data collection and analyses comply with privacy protection requirements and that no patient identification information was exposed during the study. The original contributions presented in this study are included in the article/Supplementary Materials. Further inquiries can be directed to the corresponding author.

## Supplementary Materials

The following supporting information can be downloaded at: https://www.mdpi.com/article/10.3390/curroncol32030148/s1.

## Abbreviations

HER2, human epidermal growth factor receptor 2; NSCLC, non-small-cell lung cancer; IHC, Immunohistochemistry; FISH, Fluorescence in Situ Hybridization; NGS, next-generation sequencing; ORR, objective response rate; DCR, disease control rate; PFS, progression-free survival; OS, overall survival; TRAEs, treatment-related adverse events.
